# 263 Bundling of Antimicrobial Central Venous Catheters and Interdisciplinary Collaboration to Reduce CLABSI Rates in Community Settings

**DOI:** 10.1017/ash.2026.10629

**Published:** 2026-06-23

**Authors:** Ruby Massey, Baraka Muvuka, Jonathan Guerrero, Joshua Mangum, Kyle Gospodarek

**Affiliations:** 1 Indiana University School of Medicine

## Abstract

**Background:** As of 2024, sexually transmitted infections (STI) cases remain 13% higher than they were a decade ago. Budget cuts affected 61.5% of local health department STD clinics, with 14.1% reducing clinic hours. Between 2008-2010 and 2011-2013, STI-related Emergency Department (ED) visits increased by 38.6% compared to 2% for all diagnoses. Reduced public STI funding and services have increased ED utilization. This study examined associations between social determinants of health (SDOH) and STI-related ED utilization. **Methods:** This retrospective cross-sectional study analyzed data from ED-based STI testing at three urban hospitals in Northwest Indiana between January 2021-March 2025. The dependent variable was STI-related ED visit. Independent variables were demographics, SDOH, behavioral, and health outcomes. Descriptive, bivariate (Chi-Square, Mann-Whitney U, Kruskal Wallis; p<0.05) and multivariate (Binary Logistic Regression; p<0.05) analyses were conducted using SPSS v31.0. **Results:** Conclusions: Specific SDOH are associated with STI-related ED utilization. Improving funding of publicly funded STI clinics can help to reduce healthcare costs and barriers disproportionately affecting marginalized patient populations. Future efforts should focus on strengthening ED-based SDOH screenings and referrals which may support efforts to redirect patients to cost-effective healthcare settings (e.g., public STI clinics), improving outcomes and reducing system burden.

